# Florida sleeve procedure for type II aortic regurgitation with aortic root enlargement: a case report

**DOI:** 10.1186/s44215-024-00130-0

**Published:** 2024-02-21

**Authors:** Yutaro Matsuno, Shigeru Ikenaga

**Affiliations:** Division of Cardiovascular Surgery, Department of Cardiovascular Surgery, Tokuyama Central Hospital, Yamaguchi, Japan

**Keywords:** Aortic regurgitation, Aortic root enlargement, Aortic valve plasty, Augmentation, Florida sleeve procedure

## Abstract

**Background:**

Florida sleeve procedure is an operative technique for aortic root reconstruction that offers advantages such as reduced bleeding risk, shorter operation time, and improved formation of the atrioventricular junction (AVJ) and sinotubular junction (STJ). In our department, we perform a Florida sleeve procedure for aortic regurgitation (AR) associated with aortic root enlargement of less than 40 mm in diameter of the Valsalva sinus. Here, we present a case of severe type II AR with aortic root enlargement where we successfully performed Florida sleeve procedure and augmented it with autologous pericardium.

**Case presentation:**

A 62-year-old male patient was referred for cardiovascular surgery after a transthoracic echocardiogram indicated left ventricular enlargement and severe AR. Preoperative multidetector computed tomography (MDCT) revealed AVJ of 28.2 mm, Valsalva sinus of 38.4 mm, STJ of 36.1 mm, and ascending aorta of 40.1 mm, indicating enlargement from the aortic root to ascending aorta. Preoperative transesophageal echocardiography (TEE) revealed that the main cause of AR was suspected to be the right coronary cusp prolapse (RCC). Intraoperative findings showed that the tricuspid aortic valve had no limitation of motion, but the RCC was subjected to central bending and prolapsed. The leaflet bend was thickened and shortened causing AR (type II). The geometric height (gH) of RCC was short at 14 mm, while the other valve cusps were 20 mm. An augmentation of RCC was performed using autologous pericardium, followed by a Florida sleeve procedure performed using 26-mm Gelweave Valsalva™ grafts. The gH of RCC after augmentation was 23 mm, and the effective height was adjusted to 10 mm by central plication, showing no AR by TEE. Postoperative MDCT revealed AVJ of 22.2 mm, Valsalva sinus of 30.9 mm, and STJ of 21.9 mm. Therefore, the Florida sleeve procedure provided a reduction that preserved the geometry of the aortic root, including AVJ and STJ, as intended.

**Conclusions:**

Florida sleeve procedure is a reliable and simple method for ensuring uniform aortic root geometry. A favorable outcome was obtained using the Florida sleeve procedure and augmentation with autologous pericardium in a patient with type II AR and aortic root enlargement.

## Background

Currently, the Yacoub (remodeling) and David procedures (reimplantation) are the most common valve-sparing aortic root reconstruction procedures, with significant advantages, including the avoidance of warfarin use and favorable long-term results [1, 2]. Florida sleeve procedure is a valve-sparing aortic root reconstruction technique first reported by Hess et al. [3]. The procedure does not require coronary artery reconstruction, minimizing the risk of bleeding and shortening the operation time. The procedure simply and reliably improves aortic regurgitation (AR) by comprehensively narrowing the atrioventricular junction (AVJ) and sinotubular junction (STJ). In our department, the Florida sleeve procedure is an option for AR cases associated with aortic root enlargement where the sinus of Valsalva diameter is ≤ 40 mm. We report a case of severe type II AR with aortic root enlargement that was successfully improved using the Florida sleeve technique combined with an augmentation using autologous pericardium.

## Case presentation

**Fig. 1:**
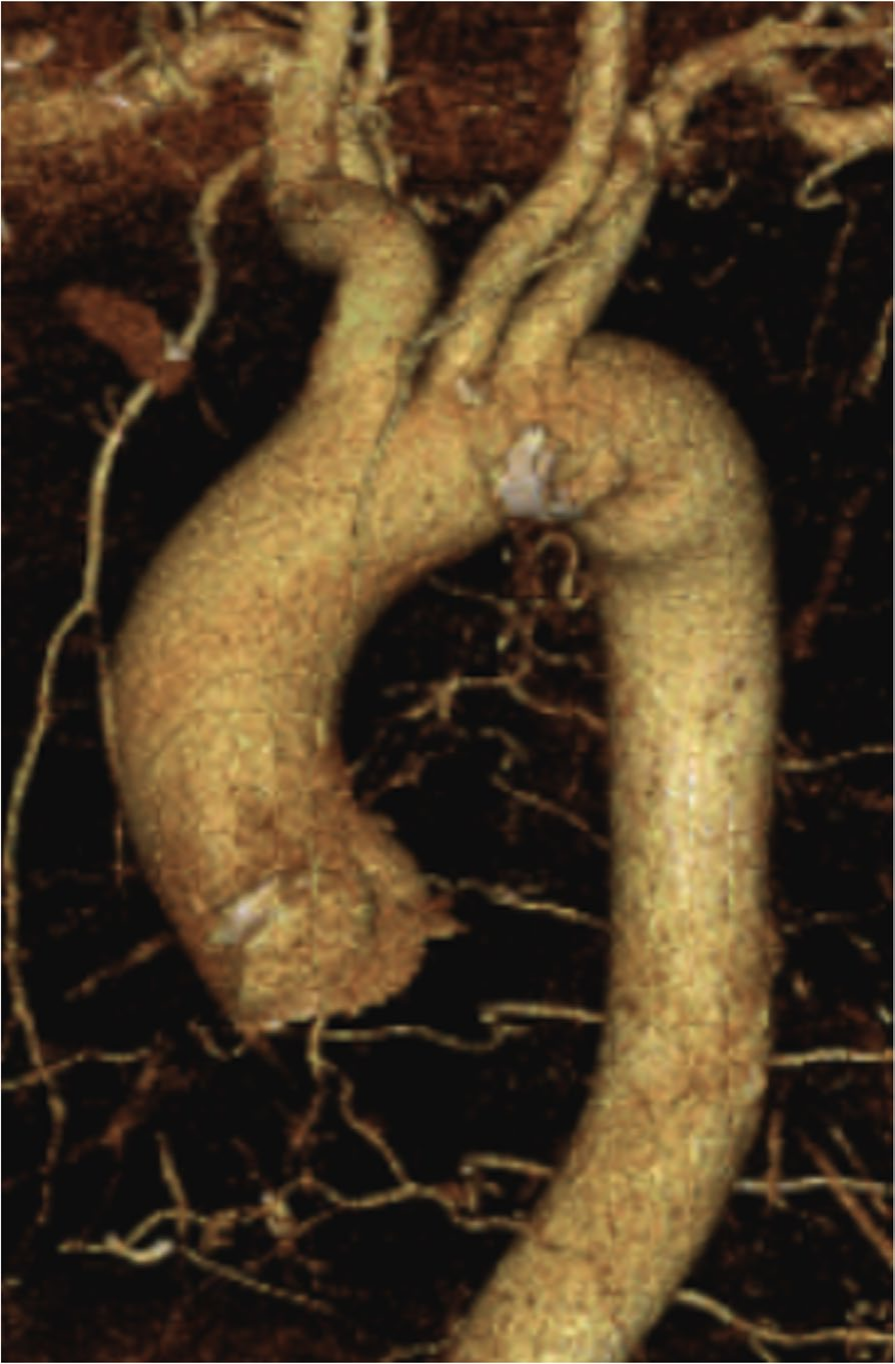
Preoperative MDCT. Preoperative MDCT examination showing AVJ of 28.2 mm, Valsalva sinus of 38.4 mm, STJ of 36.1 mm, and ascending aorta of 40.1 mm, indicating aortic root to ascending aorta enlargement. The tricuspid valve geometric height at RCC, LCC, and NCC was 10.5 mm, 17.0 mm, and 17.0 mm, and free margins at RCC, LCC, and NCC were 30.4 mm, 29.4 mm, and 30.9 mm, respectively

**Fig. 2:**
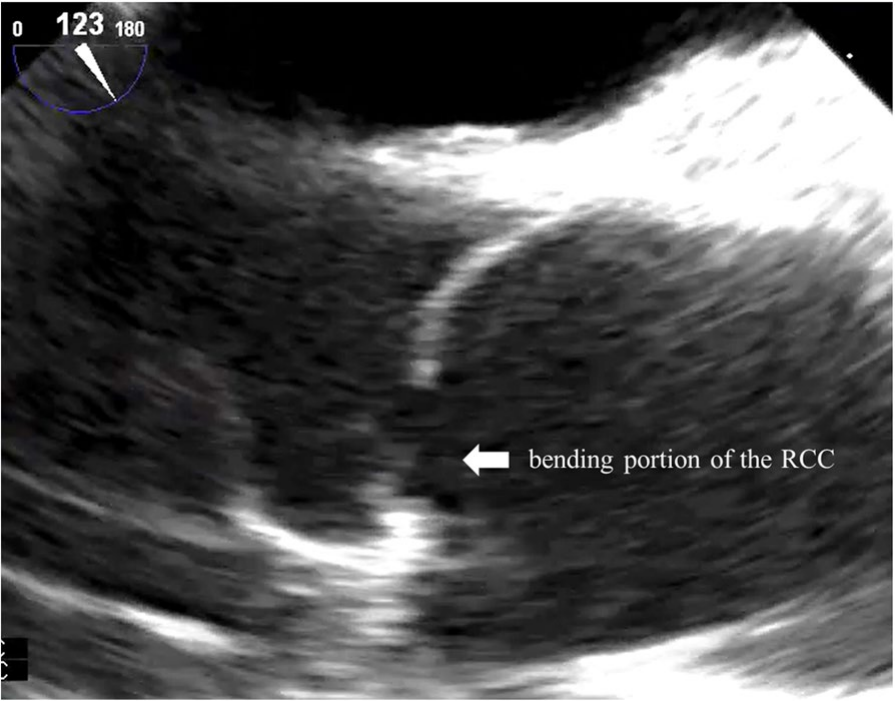
Preoperative transesophageal echocardiography images. Preoperative transesophageal echocardiography revealing that the main cause of AR was suspected to be RCC prolapse (type II)

The patient was a 62-year-old man who presented with a heart murmur noted during a physical examination 7 years earlier. The patient had a height of 161 cm, a weight of 60 kg, and a body surface area of 1.63 m^2^. A transthoracic echocardiogram (TTE) showed moderate AR, and the patient was under outpatient observation. Follow-up TTE showed a left ventricular end-diastolic diameter (LVDd) of 66 mm; left ventricular end-systolic diameter (LVDs), 42 mm; and ejection fraction (EF), 59%. The AR worsened to severe, prompting surgery. Preoperative MDCT revealed an AVJ of 28.2 mm; Valsalva sinus, 38.4 mm; STJ, 36.1 mm; and ascending aorta, 40.1 mm, indicating enlargement from the aortic root to ascending aorta (Table 1). The tricuspid aortic valve had geometric height (gH) measurements of 10.5 mm for the right coronary cusp (RCC), 17.0 mm for the left coronary cusp (LCC), and 17.0 mm for the noncoronary cusp (NCC), with free margin measurements of 30.4 mm for RCC, 29.4 mm for LCC, and 30.9 mm for NCC for clarity (Fig. 1). Preoperative transesophageal echocardiography revealed that the main cause of AR was possible RCC prolapse (type II) (Fig. 2).

**Table 1:**
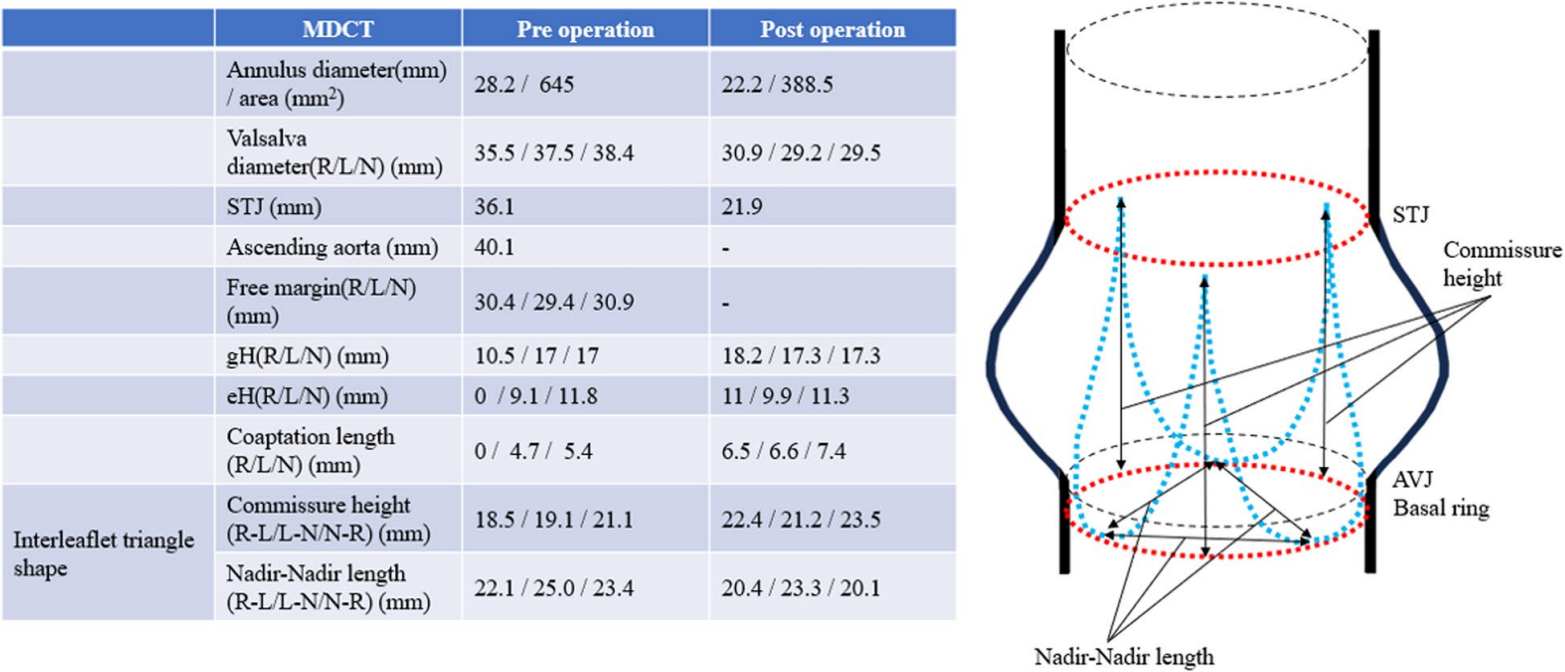
Measurements of aortic root by pre- and postoperative MDCT

The surgery was performed via a median sternotomy. Cardiopulmonary bypass was established as usual, with cannulation of the ascending aorta and superior and inferior vena cava.

**Fig. 3:**
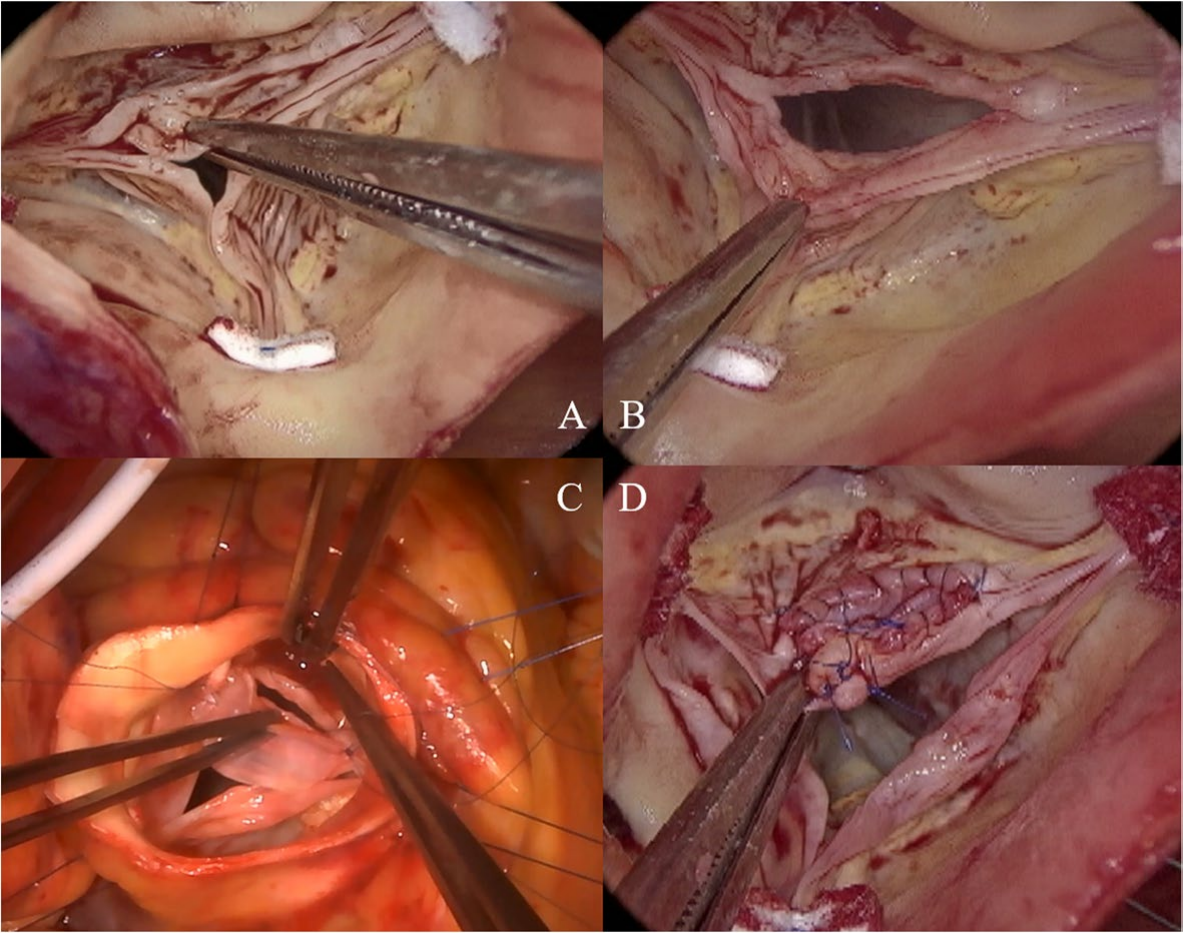
Intraoperative images of aortic valve. Intraoperative images showing the following: **A** tricuspid aortic valve with slight calcification of the RCC and NCC. The RCC presented with central bending. The leaflet bend was thickened and shortened, and the valve leaflet was prolapsed. **B** RCC with a 20-mm horizontal incision in the thickened bending area. **C** The 20 × 10 mm trimmed ovals of pretreated autologous pericardium secured to the incision site using 6-0 monofilament polypropylene sutures. **D** Increased RCC gH of 23 mm. Central plication and sutures preventing bulging were performed using 6-0 monofilament polypropylene

The ascending aorta was transected 2 cm above the ST junction, and aortic sinus and aortic valve characteristics were evaluated. The tricuspid aortic valve had slight calcifications of the RCC and NCC, but no limitation of motion. The RCC was subjected to central bending. The leaflet bends were thickened and shortened, and the valve leaflets were prolapsed, causing AR (Fig. 3A). The aortic valve measurements were as follows: gH, 14 mm, 20 mm, and 20 mm for RCC, LCC, and NCC, respectively, and effective height (eH), 0, 10 mm, and 10 mm for RCC, LCC, and NCC, respectively. A 20-mm horizontal incision was made in the thickened bending area of the RCC (Fig. 3B). Autologous pericardium was immersed in 0.6% glutaraldehyde (FUJIFILM Wako Pure Chemical Corporation, Osaka, Japan) for 5 min and then trimmed into 20 × 10 mm ovals. This was secured to the incision using running sutures with 6-0 monofilament polypropylene sutures (Ethicon, Somerville, NJ, USA) (Fig. 3C). The gH of the RCC increased to 23 mm. Additionally, central plications and sutures preventing bulging were performed with 6-0 monofilament polypropylene (Fig. 3D).

**Fig. 4:**
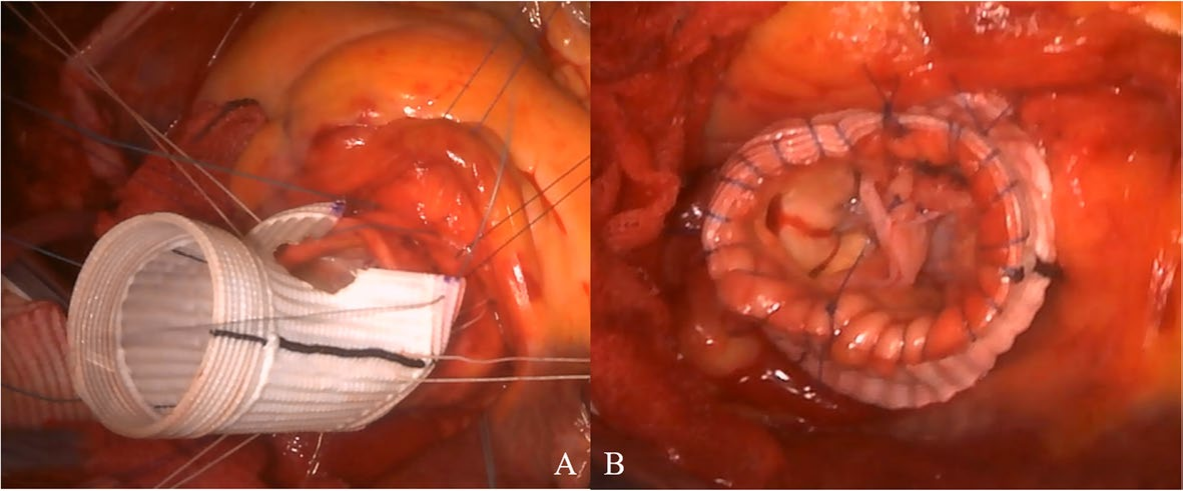
Intraoperative images of Florida sleeve procedure. Intraoperative images showing the following: **A** a hole corresponding to the size of the coronary artery in the marked area and a vertical slit between the nadir suture threads of each of the RCC and LCC on the graft creating a coronary keyhole. **B** Stump formation of the Florida sleeve procedure

**Fig. 5:**
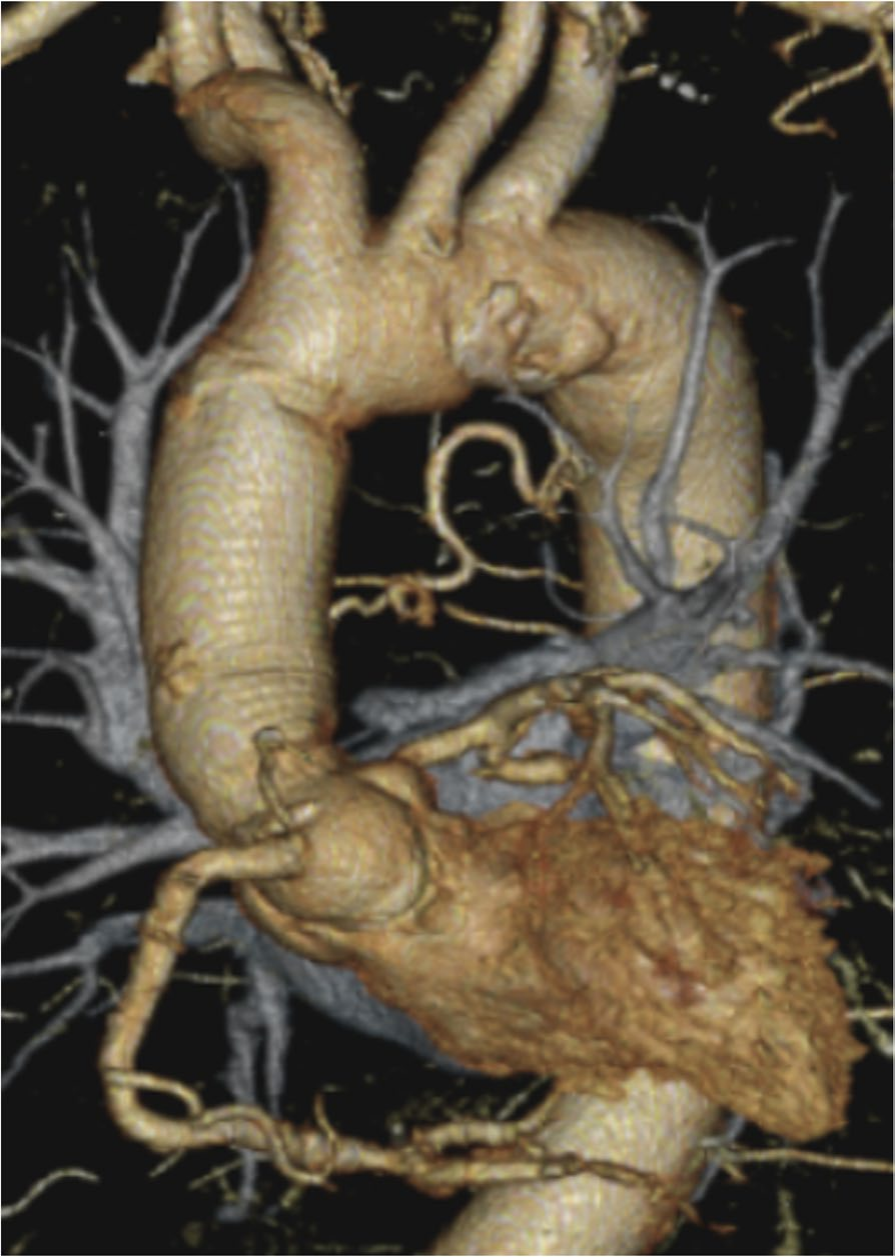
Measurements of aortic root by postoperative MDCT. Postoperative MDCT examination showing AVJ of 22.2 mm, Valsalva sinus of 30.9 mm, and STJ of 21.9 mm

The patient’s body temperature was reduced to 20 °C, and a 26-mm graft was peripherally anastomosed with the normal ascending aorta under retrograde cerebral perfusion with lower body circulatory arrest. We selected the 26-mm Gelweave Valsalva™ graft (Terumo, Ann Arbor, MI, USA) according to the gH after aortic valve repair and the assumed aortic annulus diameter. Six sub-annular horizontal mattress sutures with 4-mm spaghetti (2-0 polyester fibers; Wayolax, Matsuda Ika Kogyo Company, Tokyo, Japan) were placed beneath each of the three commissures and three nadirs at the level of the basal ring except at the R-N commissure and were driven outside the aorta. A suture at the R-N commissure was carefully passed at the level of the interleaflet triangle, positioned more distally to the basal ring. This was done with utmost caution to minimize injury risk to the membranous septum or the bundle of His. The Gelweave Valsalva™ grafts were securely affixed to the aortic root using these six mattress sutures. The graft was carefully placed as a sleeve over the aortic root, and right and left coronary artery locations were marked on the graft. A hole corresponding to the size of the coronary artery was made in the marked area, and a vertical slit was made between the nadir suture threads of each of the RCC and LCC on the graft to create a coronary keyhole (Fig. 4A). We also took special care to ensure that the coronary keyhole in the prosthesis was adequately sized to prevent any coronary artery stenosis. Each commissure was fixed to the straight part of the Valsalva graft with horizontal mattress sutures using 4–0 monofilament polypropylene sutures. Running sutures with 4–0 monofilament polypropylene were placed during the ascending aortic transection and Valsalva graft transection (Fig. 4B). The aortic valve was reevaluated. All aortic valve leaflets were confirmed to have adequate coaptation, with an eH of 10 mm. Distal anastomosis was performed between the grafts. The aortic cross-clamp time, cardiopulmonary bypass time, retrograde cerebral perfusion with lower-body circulatory arrest time, and total surgical time were 201 min, 249 min, 22 min, and 320 min, respectively. Intraoperative bleeding amounted to 340 g. Prior to commencing the operation, we collected 400 mL of autologous blood, which was subsequently reinfused after the cardiopulmonary bypass procedure. In addition, we administered 20 units of platelets intraoperatively. Transesophageal echocardiography did not reveal any AR (Fig. 5).

The postoperative course was uneventful, and TTE on the 7th postoperative day showed an LVDd of 49 mm; LVDs, 35 mm; EF, 60%; and no AR. Postoperative MDCT showed AVJ of 22.2 mm, Valsalva sinus of 30.9 mm, and STJ of 21.9 mm (Table 1). TTE showed retention of mild AR, reduction in LVDd, and prevention of re-expansion of the aortic annulus for 2 years postoperatively (Table 2).

**Table 2:**
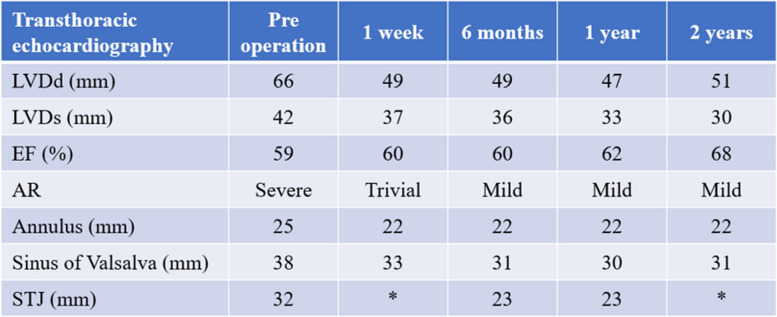
Results in transthoracic echocardiography

## Discussion

In this case, AR was mainly of type II, but the effects of type I were also considered. Florida sleeve procedure provided a reduction that preserved the geometry of the aortic root, including AVJ and STJ, as intended. The bending portion of the RCC was thickened and shortened, necessitating the extension of the valve leaflet. The gH measured intraoperatively after the aortic valve repair was more than 20 mm, which was long enough, 22 mm was targeted as the diameter of the aortic annulus after the sleeve technique with the 26 mm Valsalva graft, and the eH was determined to be retainable at 8 mm or more [4].

Hess et al. reported the Florida sleeve procedure as a simple approach for valve-sparing aortic root reconstruction for functional type I AR associated with aortic root aneurysm [3]. The procedure does not require coronary artery reconstruction, minimizing the risk of bleeding and shortening the operation time [3]. The double annuloplasty technique may also be feasible for aortic root sizes such as in this case [5]. Restoring the ratio between the aortic annulus and STJ is important for long-term repair durability [6, 7]. Compared to the double annuloplasty technique, the advantage of the Florida sleeve procedure is the possibility of maintaining a uniform and highly reproducible geometry of the aortic root. In the case involving the utilization of the 26-mm Valsalva graft, we observed reductions in dimensions, resulting in an AVJ diameter of approximately 22 mm, a Valsalva sinus measuring 30 mm, and an STJ dimension of 22 mm. The commissure was suspended and fixed at a commissure height of approximately 22 mm. Florida sleeve procedure is a reliable and straightforward technique that effectively improves AR by narrowing AVJ and STJ, thereby comprehensively shrinking the aortic root. Originally, graft selection depended on the diameter of the sinus of Valsalva, wherein 32 mm or 34 mm Dacron grafts were chosen when the diameter was dilated to 36 mm or more and 26–32 mm Dacron grafts were selected when the diameter was slightly dilated [3]. Gelweave Valsalva™ grafts should be selected as follows: 30 mm for a Valsalva sinus diameter of 5 cm or less, 32 mm for 5–6 cm, and 34 mm for 6 cm or more [8]. However, if the prosthesis size is inappropriate for the sinus of Valsalva diameter, there are concerns about residual AR and coronary artery compression due to changes in aortic root geometry. Therefore, we consider the Florida sleeve procedure to be an appropriate method of valvuloplasty in AR patients with mild enlargement of the sinus of Valsalva up to 40 mm in diameter.

In 2009, early results of the Florida sleeve procedure were reported, demonstrating persistent reductions in LVDd and LVDs and good control of AR at 3 years postoperatively [9]. Although some differences in the postoperative mechanical properties of the Florida sleeve procedure compared to remodeling and reimplantation exist, no significant impairment in valve leaflet durability has been detected at an early stage [10, 11]. AR control in the early and late periods is comparable, with a slight trend towards a slightly better survival rate in the reimplantation method compared to the Florida sleeve procedure in the late period [12]. Since the ascending aorta is 40 mm, that alone is not an indication for surgery in this case. To ensure STJ reduction when performing the Florida sleeve procedure, we use a two-piece prosthesis and perform a concomitant ascending aorta replacement. Additionally, our department follows a unified policy for conducting anastomosis under hypothermic circulatory arrest and retrograde cerebral perfusion during distal anastomosis in ascending aortic replacement surgery.

The AR in this case had type II factors and required valve leaflet extension. The options for the patch of valve augmentation include bovine pericardium and autologous pericardium. The freedom from reoperation at 10 years is 68 ± 9% for bovine pericardium patch and 72 ± 6% for autologous pericardium patch in patients in their 20s and 30s [13]. Although the current long-term results of pericardial reconstruction of valve leaflets might be inferior to valve replacement, there are advantages to preserving the autologous valve, instead of using bioprosthetic valves, which become important when considering future reoperations and transcatheter aortic valve implantation.

## Conclusions

Florida sleeve procedure is a reliable, simple, and reproducible method for ensuring uniform root geometry from AVJ to STJ. In cases of severe aortic root enlargement, there is concern about coronary stenosis and residual AR due to distortion of the coronary artery orifice and aortic wall. As a general principle, our department employs a Florida sleeve procedure for AR with aortic root enlargement of up to 40 mm diameter of the sinus of Valsalva. The present report highlights that favorable treatment outcomes can be obtained by using the Florida sleeve technique and augmentation with autologous pericardium for the management of type II AR and aortic root enlargement.

## Data Availability

The datasets during and/or analyzed during the current study are available from the corresponding author upon reasonable request.

## Abbreviations

AVJ: Atrioventricular junction
STJ: Sinotubular junction
AR: Aortic regurgitation
TTE: Transthoracic echocardiogram
MDCT: Multidetector computed tomography
LVDd: Left ventricular end-diastolic diameter
LVDs: Left ventricular end-systolic diameter
EF: Ejection fraction
RCC: Right coronary cusp
LCC: Left coronary cusp
NCC: Noncoronary cusp
gH: Geometric height
eH: Effective height
